# Advances in Functional Vascular Stents for Cardiovascular Therapy with Drug Delivery and Computational Design

**DOI:** 10.3390/pharmaceutics18070880

**Published:** 2026-07-17

**Authors:** Xiaotian Xu, Yan Hu, Haifang Li, Yu Wang, Jiayi Sun, Qiang Liu, Kairong Qin

**Affiliations:** 1 Central Hospital, Dalian University of Technology, Dalian 116089, China; 2 Faculty of Medicine, Dalian University of Technology, Dalian 116024, China; 3 School of Software Technology, Dalian University of Technology, Dalian 116024, China; 4 Department of AI and Informatics, Mayo Clinic, Jacksonville, FL 32224, USA

**Keywords:** computational design, drug delivery, hemodynamics, vascular stents

## Abstract

Vascular stents are crucial devices in the treatment of cardiovascular diseases, and their structural design and function critically affect therapeutic efficacy and patient prognosis. Conventional stents can effectively restore vascular patency by providing mechanical support to blood vessels. However, they still face significant challenges including restenosis, thrombosis, and limited adaptability to complex patient-specific lesion characteristics. To address these limitations, drug delivery offers an important strategy to modulate the pathological microenvironment, enhance long-term vascular healing, and reduce systemic side effects. Meanwhile, advances in computational simulations have provided powerful tools for optimizing stent design through structural mechanics, hemodynamics, and drug release modeling. Computational approaches enable the rational design of stent architectures with improved mechanical stability, vascular compatibility, and therapeutic regulation. Consequently, the development of vascular stents is evolving toward the synergistic integration of drug delivery, structural optimization, and intelligent design. This review summarizes the latest advances in functional vascular stents, clinical applications and computational design. This work aims to provide valuable insights for the engineering of efficient, precise, and intelligent vascular stents for cardiovascular therapies.

## 1. Introduction

Cardiovascular disease remains one of the leading causes of death and disability, with stenotic and structural abnormalities frequently involving the coronary arteries, peripheral arteries and aorta, all of which commonly require revascularization interventions. Percutaneous vascular intervention, particularly vascular stent implantation, is a key treatment for these diseases. Over decades of development, the materials and design of vascular stents have undergone several significant iterations, leading to continuous improvements in clinical efficacy. First-generation bare-metal stents (BMS) successfully addressed elastic recoil following balloon angioplasty alone, yet they were limited by high rates of in-stent restenosis (ISR) [1,2]. Subsequently, drug-eluting stents (DES) emerged as a major advancement which could significantly reduce ISR using antiproliferative drug coatings. However, the risk of late stent thrombosis (ST) remained a concern [3,4]. Dual antiplatelet therapy (DAPT) remains the cornerstone of thromboprophylaxis, yet its efficacy is limited by patient non-adherence, variable drug response, and bleeding risks with prolonged use. These limitations cannot be fully resolved by pharmacological management alone. More recently, bioresorbable scaffolds (BRS) have been developed to provide temporary mechanical support followed by gradual degradation, thereby mitigating the long-term risks associated with permanent metallic implants [5,6]. This progressive evolution of stent technology reflects a broader transition in biomaterials from non-degradable metals to biodegradable metals and polymers. Despite these advances, conventional stents remain prone to post-implantation complications such as ISR and late thrombosis. To address these challenges, drug delivery has emerged as an important strategy in next-generation stent design [7,8]. By incorporating therapeutic agents, stents can regulate the local pathological microenvironment [9], extend the duration of therapeutic action [8] and lower systemic side effects [10], thereby significantly improving biological responses and long-term efficacy. To effectively implement these delivery functions, various carriers, including hydrogels, fibers, nanoparticles, and microspheres, have been incorporated into vascular stents. These carriers not only further mitigate adverse effects and enhance localized biological response but also provide a critical technological framework for the precision release of therapeutic agents [10,11]. For the design of drug delivery in vascular stents, computational simulation plays a key role to optimize vascular stents [12,13,14,15]. In the structural design phase, simulations can evaluate radial support, flexibility, and expansion uniformity of the stent, facilitating rational optimization of mechanical performance [16,17]. In hemodynamics analysis, simulations can model local blood flow changes after stent implantation [18]. Simulations can also clarify the relationship between these changes and restenosis [19]. In studies of therapeutic agent delivery, simulation approaches allow quantitative prediction of kinetic mechanisms of agent release from carriers [20]. They also show how agents transport through the vessel wall and bind to tissues [14,21]. Collectively, these computational strategies provide a powerful framework for integrating mechanical, hemodynamic, and biological considerations in stent development.

**Figure 1:**
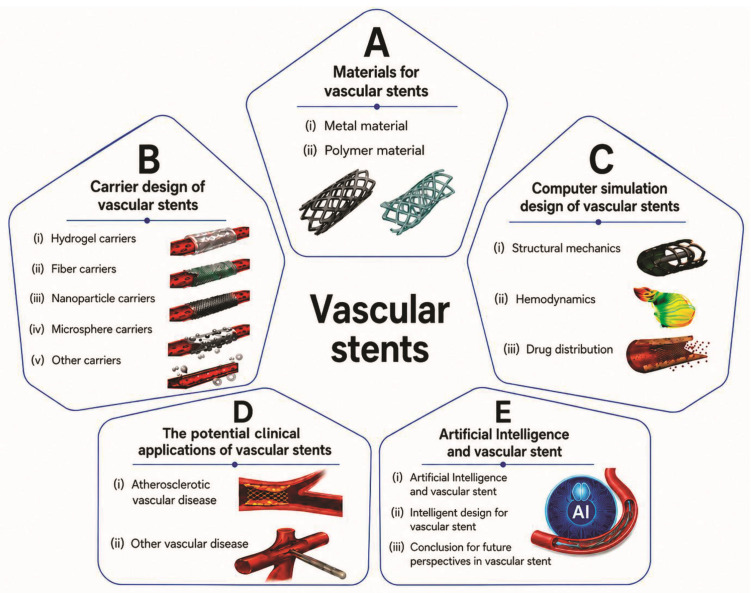
Summary of the full text logic. (**A**) Materials for vascular stents, including metallic and polymeric materials that provide the structural foundation for vascular stents. (**B**) Carrier design of vascular stents, highlighting representative carrier systems for localized therapeutic delivery and functional regulation. (**C**) Computational simulation design of vascular stents, including structural mechanics, hemodynamic analysis, and drug distribution simulation for design optimization. (**D**) Potential clinical applications of vascular stents, summarizing representative applications in atherosclerotic and other vascular diseases. (**E**) Future perspectives, emphasizing the integration of artificial intelligence with vascular stent design and the future development of intelligent vascular stents.

In recent years, the rapid development of artificial intelligence (AI) has provided new solutions for the application and research of vascular stent development, including stent material selection, carrier design, simulation optimization, and clinical decision-making. In this context, vascular stent technology is progressively evolving from mechanical implant toward an integrated therapeutic system that combines structural support of material, controlled drug delivery, intelligent design and potential clinical application (Figure 1) [22,23,24]. Therefore, this review summarizes current research landscape in functional vascular stents, with a focus on material innovation, drug delivery carriers, computational simulation, and emerging AI-driven approaches. This work aims to provide a perspective for the development of personalized, intelligent, and precision vascular stents.

## 2. Materials for Vascular Stents

The choice of stent materials plays an important role in determining the mechanical performance [25], biocompatibility [26], degradation behavior and long-term clinical outcomes [27]. To meet the evolving clinical demands for vascular intervention, several types of stent materials have been developed and optimized. Early non-degradable bare metal stents composed primarily of non-degradable metallic alloys were designed to provide mechanical support to blood vessels [28]. However, concerns regarding long-term reactions, chronic inflammation, and the permanent presence of implants have driven the development of biodegradable metallic and polymeric stents, which offer improved biological adaptability and the potential for complete degradation over time [29,30].

Based on their material composition, stents can be broadly categorized into metallic stents and polymeric stents, each possessing distinct advantages and limitations, while Table 1 provides an overview of their representative types, key properties, and current challenges [2]. It is worth noting that this paper focuses on stent and does not include Drug-coated balloons.

### 2.1. Metallic Materials

Metallic materials were used in the early stage to fabricate vascular stents due to their excellent mechanical properties and good biocompatibility, and they continue to be widely used today [26,31]. With the advancement of manufacturing technologies, metallic stents can be classified into non-degradable and biodegradable metallic stents according to their degradability [2].

#### 2.1.1. Non-Degradable Metals

Non-degradable metallic stents are the earliest class of vascular stents used in interventional therapy and remain widely applied today [32,33]. The initially developed devices were uncoated bare-metal stents [34]. These stents are primarily manufactured from stainless steel (SS), cobalt-chromium (Co-Cr), platinum-chromium (Pt-Cr), and Nitinol [34,35]. They offer distinct advantages, including robust mechanical strength, reliable radial support, and excellent biocompatibility at a low cost [34,35,36]. Stainless steel served as the pioneering material for BMS. Its corrosion resistance and mechanical stability have facilitated its long-term clinical application [34,37]. However, the primary limitation of BMS is their permanent retention within the blood vessel. In-stent restenosis remains a major potential risk [38]. Stent implantation can disrupt the endothelialization process, thereby inducing abnormal smooth muscle cell (SMC) proliferation and extracellular matrix deposition [39]. The resulting neointimal layer may lead to in-stent lumen re-narrowing. Furthermore, the rigid structure of metal stents may hinder natural vascular remodeling and endothelialization [34]. They also create a compliance mismatch with the native vessel. Over the long term, this mechanical caging may permanently impair the physiological vasomotor function of the vascular wall [40]. To address the issue of in-stent restenosis with bare metal stents, multiple studies have developed drug-eluting stents. DES utilize a non-degradable metallic framework coated with antiproliferative drugs [41]. This design significantly reduces the restenosis rate [1]. For instance, representative commercial DES such as the XIENCE series (Abbott Vascular) utilize a thin-strut Co-Cr framework to deliver everolimus, providing superior mechanical conformability and significantly lowering thrombosis rates in clinical settings [2]. Additionally, stent grafts represent a critical application of metallic stents in aortic diseases. Stent grafts rely on a non-degradable metallic skeleton enveloped by a polymer membrane, and demonstrate good safety and efficacy [42]. Despite the significant clinical success of BMS, DES, and stent grafts, their fundamental characteristic remains unaltered. The non-degradable metallic framework permanently resides within the body. Consequently, these devices continue to face formidable challenges regarding long-term safety.

#### 2.1.2. Degradable Metals

To overcome the long-term risks associated with the permanent presence of non-degradable metallic stents, degradable metallic stents have been developed. These devices aim to provide essential temporary mechanical support during vascular endothelial repair and tissue remodeling [26]. Subsequently, they progressively degrade and are absorbed by the body over months to years [43]. This temporary nature eliminates the complications caused by permanent implants [44]. Currently, magnesium (Mg) alloys are a primary focus of investigation in this field [26]. Some have achieved clinical translation and have degraded in vivo into highly biocompatible products [45,46]. Mg alloy stents provide reliable radial support during the initial implantation phase [47]. Recent clinical trials have also confirmed the robust clinical safety and efficacy of commercially available degradable Mg alloy stents [48,49,50]. Beyond Mg, researchers are actively exploring novel degradable metallic stents based on zinc (Zn) and iron (Fe) alloys. Zn alloys exhibit a degradation rate that is intermediate between those of Mg and Fe [51], which is often considered to be more compatible with the desired timescale for vascular remodeling [52,53]. At appropriate physiological concentrations, zinc ions can facilitate key cellular events that are prerequisite for endothelialization, such as the proliferation and migration of endothelial cells [54]. Conversely, Fe alloys offer excellent mechanical properties, with a radial strength comparable to stainless steel [51]. However, their clinical application is hindered by a slow degradation rate [51]. Collectively, these three degradable metals present distinct advantages and limitations, offering diverse options for the advanced design of next-generation degradable stents.

Metallic vascular stents are evolving from permanent implants to degradable platforms [44]. Correspondingly, stent materials have advanced from conventional stainless steel to bioresorbable metallic systems predominantly represented by Mg alloys [55,56]. Despite these advancements, current degradable metals still possess inherent limitations. Therefore, synergistically optimizing mechanical strength and degradation kinetics remains the core challenge for the future development of degradable metallic stents [51,57].

### 2.2. Polymer Materials

Materials science and medical engineering are becoming deeply integrated. Similarly to metallic vascular stents, polymeric stents can be divided into two categories based on their degradation behavior, i.e., non-degradable and degradable. Degradable polymeric stents, made from Poly(L-Lactic Acid) PLLA, Poly(Lactic-co-Glycolic Acid) PLGA, Polycaprolactone (PCL) and other polymers, offer unique degradability, biocompatibility, and design flexibility. As a result, they have gradually become a research hotspot in the field of vascular stents. However, non-degradable polymers have significant limitations in mechanical properties and long-term biostability [2]. Their clinical application is extremely rare. Therefore, most research efforts have shifted toward degradable polymeric vascular stents.

Degradable polymeric vascular stents have achieved relatively broad clinical application, and related research has advanced considerably [29]. Their core advantage lies in complete degradation and absorption after fulfilling the mechanical support function, thereby avoiding the long-term risks associated with permanent implants and potentially restoring normal vascular vasomotion [5]. Based on chemical structure, degradable polymers can be mainly classified into polyesters (e.g., PLLA, PLGA, PCL), polyurethanes (e.g., zwitterionic polyurethane), and polycarbonates (e.g., tyrosine-derived polycarbonate) [2,58,59]. Polyesters are the most widely studied and applied biodegradable synthetic polymers, with PLLA as a representative example, constituting the main material of first-generation BRS [60]. However, first-generation BRS based on PLLA exhibited a relatively high risk of stent thrombosis in early clinical trials due to issues such as thick struts and insufficient radial strength [29,61]. A milestone clinical example is the Absorb Bioresorbable Vascular Scaffold (Abbott), which proved the feasibility of complete PLLA resorption but ultimately faced clinical challenges due to its thick struts. In contrast, the Fantom scaffold (REVA Medical) utilizes tyrosine-derived polycarbonates to achieve thinner struts and inherent radiopacity, exemplifying next-generation polymer innovations [2]. To address these limitations, subsequent studies have improved polymer processing techniques (e.g., crystallization optimization) and developed copolymers (e.g., PLGA). Polyurethane materials have been introduced as novel smart polymers for stent design, such as zwitterionic polyurethane and poly (urethane-urea) elastomers, which exhibit excellent hemocompatibility and adaptive mechanical properties [25]. Studies have shown that with process optimization and material improvements, this class of vascular stents can achieve better clinical performance. Polycarbonate materials, represented by tyrosine-derived polycarbonates, have been investigated for vascular stent applications based on L-tyrosine pseudo-poly (amino acids) and other tyrosine analogues. These materials utilize natural amino acids as building blocks, allowing for tunable backbone structures through chemical synthesis. Their advantages include excellent biocompatibility, controllable degradation, and inherent radiopacity achieved via molecular design (e.g., iodinated tyrosine rings), which facilitates clinical imaging follow-up. The potential of such materials to improve radial strength and reduce strut thickness has been well-validated [2,62]. Meanwhile, the emergence of additive manufacturing (3D/4D printing) technologies has provided new possibilities for the design and fabrication of synthetic polymer stents. Three-dimensional printing facilitates the fabrication of stents with complex topological geometries to optimize mechanical characteristics while allowing for patient-specific customization, and such well-designed geometric configurations are capable of improving the local haemodynamic environment [46,63,64]. Furthermore, 4D printing has been explored to achieve a milder, more conformable self-expanding process, thereby reducing vascular wall injury caused by dilation [65]. Shape memory polymers have been confirmed as one of the key materials for 4D printing of vascular tissue-related constructs [66]. In addition to these synthetic polymers, natural polymers such as silk fibroin, collagen, and chitosan offer superior biocompatibility, low immunogenicity, and the capacity to promote cell adhesion and proliferation, making them attractive candidates for vascular repair [67,68,69,70,71]. These materials offer superior biocompatibility, low immunogenicity, and the capacity to promote cell adhesion and proliferation [67,69,72]. While less commonly used for stents, natural polymers have been extensively investigated for tissue-engineered vascular grafts [73,74,75]. Researchers utilize techniques such as electrospinning, freeze-drying, and 3D bioprinting to fabricate grafts for the replacement of diseased vessels. These constructs eventually remodel into functional vascular tissues in vivo. Although these studies do not focus directly on stent design, they provide innovative strategies for vascular restoration.

In summary, this section outlined the evolution of stent materials from non-degradable metals to biodegradable metals and polymers. The following section shifts the focus to how carrier systems can complement these bulk materials by imparting additional therapeutic functions.

## 3. Carrier Design of the Vascular Stent

**Figure 2:**
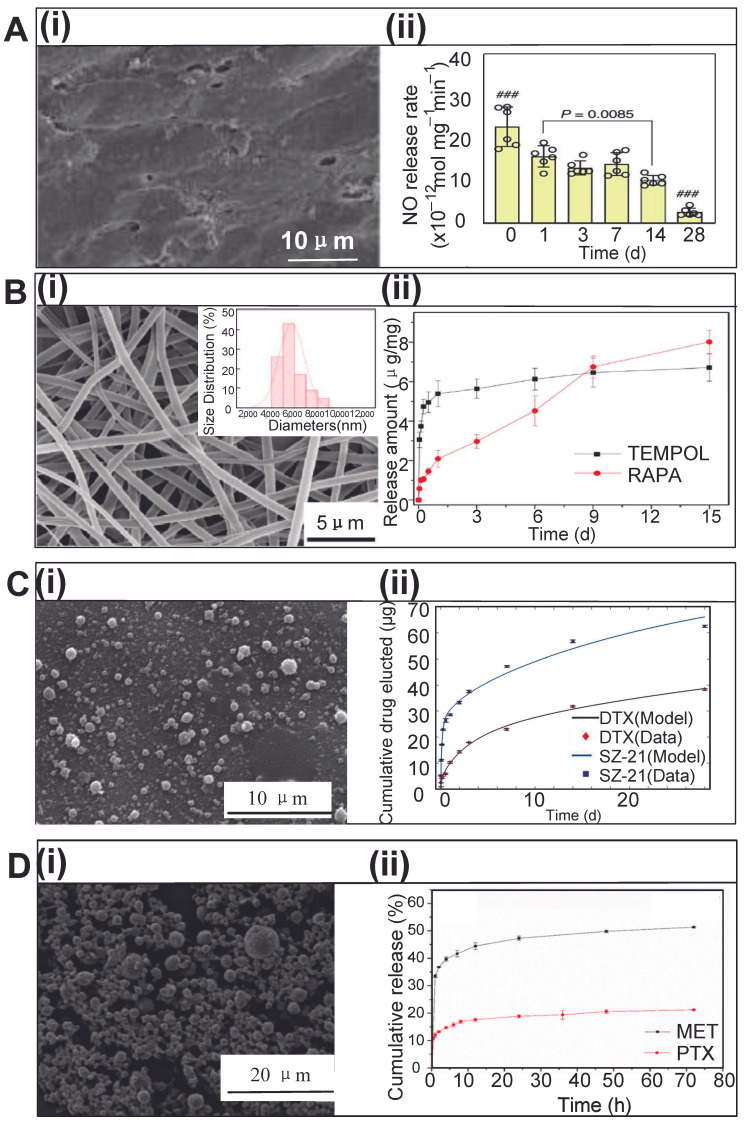
Microscopic morphologies and controlled release kinetics of four typical stent-based carriers. (**A**) Hydrogel: (**i**) Scanning electron microscopy (SEM) image of the NO-eluting hydrogel coating after in vitro balloon dilation. (**ii**) In vitro NO release over 28 days, ### means *p* < 0.001 compared with other groups [82]. (**B**) Fiber: (**i**) SEM image of the electrospun nanofiber membrane co-loaded with rapamycin and TEMPOL. The inset shows the statistical histogram of the corresponding nanofiber diameter distribution. (**ii**) In vitro cumulative release of rapamycin and TEMPOL over 15 days [83]. (**C**) Nanoparticle carriers: (**i**) SEM image of the morphology of the hydrophobic core/hydrophilic shell nano/micro particles used as a drug-eluting stent coating [84]. (**ii**) In vitro cumulative release profiles of DTX and SZ-21 from the core/shell particle-coated stent. (**D**) Microsphere carriers: (**i**) SEM image of SF-PTX-MET-MT microspheres for stent modification. (**ii**) In vitro cumulative release of PTX and MET from the coated stent over 70 h [85]. All images are reproduced under the Creative Commons Attribution License.

While contemporary bulk materials have made significant advancements in mechanical support and degradation control [2,26], they alone are insufficient to meet the complex biological requirements of vascular repair [11,76]. These sophisticated demands include anti-proliferation [77], pro-endothelialization [78], anti-thrombosis [79], and temporal regulation [80]. Carrier systems can load and protect various therapeutic agents, control their release in a spatiotemporal manner, actively remodel the local pathological microenvironment, and promote endothelial cell proliferation [11,81]. Therefore, the incorporation of carriers into stent systems can significantly expand their therapeutic functions. Figure 2 illustrates the microstructure and release kinetics of four typical stent-based carriers [82,83,84,85], accordingly, this section focuses on their design strategies. The performance comparison of various carriers is shown in Table 2.

**Table 2:** Comparative analysis of functional carrier platforms for stent-based therapeutic delivery.

Carrier Type	Payload Types Supported	Typical Release Duration	Key Advantages	Main Limitations	Translational Maturity
Hydrogels	Small molecules, Growth factors, Genes, Gases	Days to weeks (e.g., 17–28 days)	3D hydrophilic network, tunable degradation, supports synergistic multi-drug release	Insufficient mechanical strength for bulk use	Preclinical in vivo; clear path for functional coatings
Fibers	Small molecules, Growth factors	Up to ~30 days	High surface area, ECM-mimetic, can serve as structural stent body	Requires design to maintain kinetics during expansion	Preclinical in vivo; advanced proof-of-concept for dual-release
Nanoparticles	Small molecules, Genes, Gases, Cells	Up to 4–8 weeks (e.g., 60 days)	Subcellular delivery, active/stimuli-responsive targeting	Complex cross-linking or matrix required for attachment	Advanced preclinical in vivo (porcine/rabbit models)
Microspheres	Small molecules, Biologics, Gases, Cells	Days to weeks (e.g., 28 days)	High loading capacity, programmable release, can function as embolics	Typically requires embedding in hydrogels or dopamine-mediated attachment	Preclinical in vivo; emerging clinical utility in embolization
Liposomes & Exosomes	Hydrophilic/hydrophobic drugs, Genes	Varies	Overcomes poor solubility (Liposomes); immunomodulation (Exosomes)	Biostability and scalable manufacturing challenges	Early preclinical in vivo (proof-of-concept)
Viral Vectors	Genes (e.g., AAV, Adenovirus)	Varies	Extremely high gene transduction efficiency	Complex anchoring strategies needed for stent surfaces	Early preclinical in vivo (proof-of-concept)

The design of drug-eluting stent carriers begins with understanding the pharmacological properties of the therapeutic agent. Two classes dominate DES: the limus family (cytostatic, mTOR inhibition, binding-dominated transport) and paclitaxel (cytotoxic, microtubule stabilization, convection-dominated transport). These differences in mechanism of action, tissue binding, and transport behavior directly dictate carrier selection and release kinetics [86]. A comparative overview is provided in Table 3.

### 3.1. Hydrogel Carriers

Hydrogels possess unique three-dimensional hydrophilic networks, excellent biocompatibility, tunable degradation behavior, and mild drug-loading conditions, rendering them highly suitable for the functional modification of vascular stents [87]. Some studies suggest that hydrogels are unsuitable as bulk materials for vascular stents due to their insufficient mechanical strength and inability to maintain luminal patency [88]. Although recent studies have preliminarily validated the feasibility of hydrogels as bulk vascular implants [89], to our knowledge, structural hydrogel carriers remain at the stage of proof of concept. In contrast, functional hydrogel carriers have made substantial progress, presenting a clearer path toward clinical translation. The core mission of these carriers is to endow stents with active delivery capabilities. Acting as reservoirs for bioactive molecules, they enable the precise delivery of therapeutic agents to target sites and facilitate local intervention through hydrogel swelling, diffusion, or degradation [90,91,92]. Furthermore, hydrogel carriers enable targeted drug delivery. One study used thermosensitive Pluronic F-127 hydrogel as a rapamycin carrier. By tuning the porosity of porous NiTi alloy (0–40%), it achieved precise control over drug loading and sustained release for up to 17 days, effectively inhibiting smooth muscle cell (SMC) proliferation [93]. Building on this, another study developed a bilayer coating composed of rapamycin-loaded PLGA and heparin-loaded alginate hydrogel. The coating enabled the release of both agents from the stent surface, achieving synergistic anti-proliferative and anticoagulant effects [94]. In addition, an asymmetric coating strategy was employed: a heparin-loaded alginate hydrogel coating and an atorvastatin calcium-loaded polyurethane electrospun fiber coating were fabricated on coronary stents. This dual-drug release design provided multimodal therapeutic benefits [95]. These advances demonstrate that hydrogel drug delivery systems are evolving from single-agent release toward multi-drug synergy and spatial control. Hydrogel carriers also support growth factor delivery. One study encapsulated human vascular endothelial growth factor-165 in calcium alginate microspheres, which were then dispersed with paclitaxel-loaded micelles in a polyvinyl alcohol (PVA) hydrogel matrix. This system achieved rapid short-term VEGF release to promote early endothelialization, while sustained long-term paclitaxel release inhibited late SMC proliferation. This combinatorial delivery approach may better match the stage-specific therapeutic requirements of vascular injury repair [96]. Hydrogel carriers are capable of gene delivery as well. Cationized gelatin hydrogels have been shown to effectively deliver LacZ plasmid DNA and achieve sustained in vivo gene expression, confirming the fundamental feasibility of hydrogel-mediated non-viral gene delivery [97]. Subsequently, a cationized pullulan hydrogel delivered siRNA targeting matrix metalloproteinase 2 (MMP-2), resulting in reduced pro-MMP-2 activity [98]. Further work constructed a fibrin hydrogel gene reservoir via LbL self-assembly, which loaded and protected VEGF/Ang1 gene nanoparticles. In vivo experiments confirmed that this strategy significantly enhanced re-endothelialization [99]. These developments illustrate the progression of hydrogel gene delivery systems from proof-of-concept to therapeutic application. Additionally, hydrogel carriers enable gaseous molecule delivery. One study developed a mechanically robust nitric oxide (NO)-eluting hydrogel coating (Figure 2A) [82], which continuously generated NO by catalyzing endogenous substrates. In both rabbit and porcine stent models, this coating effectively suppressed local inflammation, accelerated re-endothelialization, and attenuated neointimal hyperplasia [82]. Furthermore, a hydrogel coating with sequential H_2_S/NO release was fabricated: an early H_2_S burst synergized with NO to inhibit thrombosis and inflammation, while sustained NO release maintained vascular homeostasis, leading to superior endothelialization and reduced intimal hyperplasia [100]. Additional gas delivery strategies have also been reported: a catechol-chitosan hydrogel coating loaded with Cu^2+^ catalyzed endogenous NO production [101], and a catechol-hyaluronic acid-cystamine hydrogel coating loaded with allicin (an H_2_S donor) achieved oxidative stress-responsive on-demand release [102]. These examples highlight the tremendous potential of hydrogels in gas therapy for cardiovascular applications.

In summary, while hydrogels excel in offering mild loading conditions and highly tunable 3D networks for synergistic multi-agent delivery (e.g., drugs, genes, and gases), their independent clinical translation remains bottlenecked. The critical unresolved challenge lies in resolving the inherent trade-off between maintaining sufficient hydrogel swelling for diffusion-controlled release and ensuring robust interfacial adhesion to the metallic stent strut to prevent coating delamination under high arterial shear stresses. Moreover, their insufficient mechanical strength restricts their use as bulk structural materials, confining them primarily to functional coatings.

### 3.2. Fiber Carriers

Fiber materials are widely employed as carriers for vascular stents due to their high specific surface area, high porosity, and fibrous network that effectively mimics the natural extracellular matrix [103]. Furthermore, these materials offer considerable flexibility in tailoring mechanical properties and degradation kinetics through material selection and fabrication process design [104,105]. Structural fiber carriers make use of fibers to directly form the stent body, maintaining luminal patency through their inherent mechanical properties [106]. While these polymeric materials, including PLLA, PCL, and PVA, were introduced from a material science perspective in Section 2.2, this section focuses specifically on their application as the primary structural components of stents. Beyond their role as functional coatings, structural fibers can also be engineered into the stent architecture itself. For instance, Lin et al. demonstrated the benefits of this approach by weaving PCL/PEG-coated PVA yarns into a vascular stent, followed by heat treatment to create a distinct skin–core structure. By exploiting the inherent designability of woven architectures, this approach endowed the stent with superior flexibility and elasticity, effectively demonstrating the structural advantages of utilizing fiber-based carriers as the stent body [107].

Functional fiber carriers endow stents with therapeutic functions, such as targeted drug delivery and biological activity regulation. Drug delivery via fiber carriers has been extensively investigated. For example, an asymmetric coating technique was adopted to fabricate a heparin-loaded alginate layer and an atorvastatin calcium-loaded polyurethane electrospun fiber layer on coronary stents, achieving dual-drug release [95]. Another study prepared paclitaxel-loaded PCL electrospun coatings for stents and systematically investigated the influence of coating deformation during stent expansion on drug release behavior. The results revealed that coating elongation did not induce fiber fracture, and drug release kinetics remained stable. This provides an important reference for the coating design of drug-eluting stents, demonstrating the stability and controllability of fiber carriers in drug delivery [108]. To achieve multi-dimensional interventions in complex pathological microenvironments, an electrospun nanofiber-covered stent co-loaded with rapamycin and the antioxidant TEMPOL was developed (Figure 2B). The fiber carrier achieved sustained and steady co-release of both therapeutics for up to 28 days. It inhibited smooth muscle cell proliferation while effectively scavenging local reactive oxygen species (ROS), thereby significantly promoting re-endothelialization and alleviating local inflammation [83]. Beyond drug delivery, fiber carriers have also been engineered to co-deliver drugs and growth factors for vascular stent applications. For instance, a PLCL nanofiber-covered stent co-loaded with heparin and VEGF was constructed to enable sustained dual release for 30 days, facilitating endothelial cell proliferation and stent re-endothelialization [109]. Advancing this approach, another study designed PLA electrospun fiber-covered stents with spatially compartmentalized delivery: VEGF was covalently immobilized on the fiber surface, while paclitaxel was encapsulated into mesoporous silica nanoparticles embedded within the fiber matrix. This architecture enabled the rapid release of VEGF alongside the long-term sustained release of paclitaxel, achieving spatiotemporal regulation of dual-agent release [110]. These advances indicate that growth factor delivery via fiber carriers is evolving toward sophisticated drug-factor combinations with spatiotemporally regulated release profiles.

Overall, fiber carriers can accommodate both structural integrity and complex spatiotemporal dual-release profiles. However, a major unresolved engineering hurdle is managing coating deformation. While current studies indicate that specific fiber coatings can withstand elongation without immediate fracture during stent expansion, ensuring uniform deformation without altering the intended drug release kinetics or causing micro-tears in highly tortuous vascular beds remains a significant translational challenge.

### 3.3. Nanoparticle Carriers

Nanoparticles have been extensively utilized in vascular disease research and serve as highly effective carriers for vascular stents. This widespread application is driven by their unique advantages, including nanoscale size effects, high specific surface area, tunable surface chemistry, and remarkable capabilities for subcellular delivery and active targeting [111,112]. Currently, research on nanoparticles in the context of vascular stents predominantly focuses on their application as functional carriers. In this paradigm, the stent serves as a delivery platform loaded with nanoparticles to achieve therapeutic functions such as gene therapy, targeted drug delivery, and the inhibition of restenosis [113,114,115]. Nanoparticles have a wide range of applications in vascular stent technology, most notably in drug delivery. One study employed cationic electrodeposition to coat stent surfaces with bioabsorbable polymeric nanoparticles encapsulating a fluorescent marker. In a porcine coronary artery model, the resulting nanoparticle-eluting stent demonstrated sustained local drug release for up to 4 weeks without aggravating stent-induced inflammatory responses or neointimal hyperplasia [116]. Another study encapsulated sirolimus into nanoparticles and delivered them locally to the stent implantation site, confirming their inhibitory effect on in-stent restenosis in a preclinical model [117]. Similarly, pitavastatin-loaded nanoparticle-eluting vascular stents effectively inhibited in-stent restenosis without delaying endothelial healing in a porcine coronary artery model, offering new insights into overcoming the side effects of conventional drug-eluting stents [118]. Beyond the choice of therapeutic agents, the structural design of nanoparticle coatings critically influences stent performance. For instance, one study engineered hydrophobic core/hydrophilic shell nanoparticulate systems using coaxial electrospray to achieve the differential release of dual therapeutic agents (Figure 2C) [84]. By encapsulating the anti-proliferative drug docetaxel (DTX) within the core and the anti-platelet antibody SZ-21 within the shell, this precise core/shell architecture facilitated a highly controlled, biphasic drug release profile. This structural design effectively addressed both smooth muscle cell proliferation and platelet activation simultaneously. To further improve delivery efficiency, researchers have introduced targeting strategies. Chorny et al. applied magnetic targeting by encapsulating paclitaxel into magnetic nanoparticles, which, under an external uniform magnetic field, facilitated localized accumulation at the stent and significantly enhanced drug retention at the injury site [119]. They subsequently reviewed the promise of magnetic nanoparticles for targeted vascular delivery and discussed the potential of uniform magnetic field-mediated targeting strategies in preventing in-stent restenosis [115]. Furthermore, Zhao et al. constructed reactive oxygen species (ROS)-responsive nanoclusters loaded with RVX-208. Following intravenous injection, these clusters specifically dissociate and release the drug under the high-ROS environment of the arterial injury site, achieving an on-demand release profile that minimizes systemic exposure while enhancing local therapeutic efficacy [120]. Nanoparticles also enable highly effective gene delivery. Che et al. complexed Akt1 siRNA with disulfide-crosslinked low-molecular-weight polyethylenimine to form nanoparticles, which were applied onto a hyaluronic acid-coated stent surface. In vivo experiments in a rabbit model demonstrated that this approach significantly inhibited smooth muscle cell growth [113]. Building on this, the same team systematically investigated the therapeutic effect of this Akt1 siRNA nanoparticle-eluting stent on restenosis following balloon injury, further confirming the feasibility of the strategy through micro-CT and histological analyses [121]. The same group also loaded miR-145 into the identical carrier system, validating its inhibitory effect on restenosis in a rabbit iliac artery stent model [114]. Moreover, Izuhara et al. developed a miR-126-loaded nanoparticle-eluting vascular stent, demonstrating its significant suppression of neointimal formation in a rabbit model [122]. Another study utilized ultrasound-mediated magnetic nanoparticle delivery of Pik3cb shRNA and confirmed its inhibitory effect on intimal thickening in a rat balloon injury model [123]. Collectively, these studies indicate that nanoparticle-mediated gene delivery is advancing from siRNA to miRNA, from single to multiple targets, and from passive release to physical field-assisted delivery. Apart from gene delivery, nanoparticles also enable gasotransmitter delivery to achieve multifunctional stent coatings. Luo et al. constructed a surface coating loaded with heparin nanoparticles that simultaneously generates NO, achieving dual anticoagulant and pro-endothelialization functions [124]. The application of nanoparticle carriers in vascular stents has also extended to emerging areas such as immunomodulation. Li et al. developed a rapamycin-loaded, platelet membrane-camouflaged biomimetic nanoparticle-coated stent, which promoted vascular healing by modulating the cGMP-PKG and NF-κB signaling pathways [125]. Hou et al. immobilized exosomes onto the surface of biodegradable magnesium alloy stents, harnessing the natural bioactive molecules within exosomes to achieve anti-inflammatory and pro-endothelialization functions [126]. Polyak et al. employed magnetically mediated targeted delivery of superparamagnetic nanoparticle-loaded endothelial cells, demonstrating significant inhibition of in-stent stenosis in a rat carotid artery stent model [127]. These emerging strategies expand the application boundaries of nanoparticles in vascular stents and open up new possibilities for functionalized design.

Ultimately, nanoparticles offer unparalleled capabilities for subcellular targeting, stimuli-responsive release, and magnetic field-guided accumulation. Nevertheless, their integration into vascular stents remains technologically complex. Because nanoparticles rarely function as standalone continuous coatings, they heavily rely on secondary immobilization matrices including polyphenol cross-linking networks and hyaluronic acid base layers. This multi-step functionalization significantly increases manufacturing complexity and raises unresolved regulatory concerns regarding the long-term in vivo biostability of these complex composite interfaces.

### 3.4. Microsphere Carriers

Microspheres serve as highly effective carriers for vascular stents due to their core advantages, including tunable size and architecture, high drug-loading capacity, and programmable release kinetics [85,128,129]. Also acting as functional carriers, they are primarily utilized to achieve the localized and controlled release of therapeutic agents [130,131].

One primary application of microsphere carriers is drug delivery. One study embedded PLGA microspheres loaded with hydrophilic drugs into a hydrogel coating, tailoring the drug release rate by altering the polymer formulation [129]. Another study assembled silk fibroin microspheres loaded with both paclitaxel and metformin onto the surface of a woven stent-graft via electrostatic bonding. This dual-drug synergy achieved robust anti-proliferative functions, with in vitro release sustained for over 70 h (as shown in Figure 2D) [85]. Furthermore, researchers constructed salvianolic acid B-loaded chitosan microspheres on a nitinol vascular stent utilizing a dopamine-mediated strategy. Leveraging the dual bioactivity of salvianolic acid B, this microsphere structure significantly inhibited the proliferation and migration of smooth muscle cells in vitro while promoting the adhesion and proliferation of endothelial cells, offering a reliable strategy for the long-term prevention of in-stent restenosis [77]. To enhance anticoagulant function, another study immobilized heparin/poly-L-lysine microspheres onto a dopamine-coated stent surface. This approach significantly prolonged both the activated partial thromboplastin time (APTT) and thrombin time (TT), demonstrating excellent anticoagulant activity alongside promoted endothelial cell adhesion and proliferation [131]. Collectively, these studies indicate that microsphere-mediated drug delivery is evolving from single-agent formulations to dual-drug synergy, and from mono-functional to multi-functional designs. Beyond traditional pharmacotherapeutics, microspheres also facilitate the localized delivery of bioactive factors. For instance, biodegradable microspheres encapsulating NO donors have been incorporated into vascular stents. These stents achieved an initial NO burst release during the first week followed by sustained release for three weeks, significantly reducing the intima-to-media ratio by 46% and 32%, respectively, at 7 and 28 days post-implantation, indicating time-dependent vascular remodeling benefits [130]. In another study, a composite coating consisting of basic fibroblast growth factor (bFGF)-loaded gelatin hydrogels and argatroban-loaded PLGA microspheres was applied to cerebral aneurysm stents. This configuration promoted connective tissue formation within the aneurysm cavity while reducing the incidence of in-stent thrombosis [128]. This highlights that microsphere-based factor delivery is expanding from small-molecule gas therapeutics to protein-based biologics, and enables the construction of multi-agent composite coatings. Microspheres also enable advanced cell-loading strategies. One study utilized superparamagnetic iron oxide microspheres to intracellularly label endothelial cells. Through the magnetic forces generated by a magnetized stent, these cells were successfully captured and retained at the implantation site, providing a novel paradigm for accelerating stent surface endothelialization [132]. Additionally, microspheres can function directly as embolic agents. In aneurysm treatment, a recent strategy involves deploying a flow-diverting stent followed by the injection of 500–900 μm microspheres into the aneurysm sac through the stent interstices. Acting as a mechanical barrier to prevent microsphere migration, the stent facilitates safe and complete aneurysm occlusion [133]. This innovative approach broadens the role of microspheres in vascular stent-related therapies, extending their application from therapeutic delivery to interventional embolization.

In conclusion, microspheres present a robust platform characterized by high drug-loading capacity and highly programmable release kinetics extending over several weeks. They even demonstrate unique utility as physical embolic agents. However, akin to nanoparticles, the unresolved bottleneck lies in their surface immobilization. Existing strategies requiring embedding within hydrogels or electrostatic/dopamine-mediated bonding often struggle to guarantee uniform spatial distribution across the stent struts, potentially leading to uneven drug deposition within the target vascular wall, which is a critical factor for preventing localized restenosis.

### 3.5. Other Carriers

Beyond the aforementioned hydrogels, fibers, nanoparticles, and microspheres, a variety of novel carrier systems have emerged in recent years, further enriching the functional design strategies for vascular stents. Research on the application of functional carriers (e.g., liposomes, exosomes, and viral vectors) is continuously advancing, offering novel solutions for localized drug and gene delivery.

Liposomes, vesicular structures composed of phospholipid bilayers, have garnered widespread attention in vascular stent drug delivery systems due to their excellent biocompatibility, capacity to encapsulate both hydrophilic and hydrophobic drugs, and amenability to functional modification. In the realm of drug delivery, alendronate-loaded liposomes exert anti-inflammatory effects by specifically targeting monocytes and macrophages; systemic administration of these liposomes significantly inhibits neointimal hyperplasia following stent implantation [134]. Furthermore, liposomal nanocarriers can enhance the delivery efficiency of sirolimus by overcoming its poor solubility [135]. In gene delivery, complexes formed by cationic liposomes and nucleic acids within vascular stents enable the localized gene transfection of vascular tissues [136] and, liposome-functionalized stents carrying the endothelial nitric oxide synthase (eNOS) gene have been demonstrated to accelerate re-endothelialization [137].

Exosome carriers are nanoscale vesicles (30–150 nm) secreted by cells, carrying a variety of bioactive molecules such as proteins, mRNA, and miRNA, and possessing natural intercellular communication functions [138]. In the field of gene delivery, one study loaded plant-derived exosome-like nanoparticles with CA1-siRNA and achieved targeted delivery to aortic injury sites, effectively inhibiting in-stent restenosis [139].

Viral carriers hold significant value in the field of vascular stent gene therapy owing to their high gene transduction efficiency. In the context of gene delivery, one study covalently anchored Protein G to the metallic stent surface, utilizing its natural binding affinity for the Fc region of mammalian IgG to capture adeno-associated virus, thereby enabling vector-mediated gene delivery from the vascular stent [140]. while hydrolyzable linkers allow controlled viral vector release [141]. Furthermore, viral vectors can also be delivered within vascular stents using a balloon catheter; one study employed this approach to achieve local intraluminal delivery of adenoviral VEGF-A at the stent site, significantly accelerating the re-endothelialization process on the stent surface [142].

To synthesize, emerging biological vectors such as liposomes, exosomes, and viral carriers address highly specific niches, ranging from solubilizing hydrophobic drugs to achieving unparalleled gene transduction efficiencies. Yet, their translational maturity lags significantly behind synthetic polymers. Unresolved issues including strict biological storage requirements, scalable manufacturing hurdles, and the necessity for complex surface-anchoring chemistries (e.g., hydrolyzable crosslinkers) severely limit their immediate clinical viability.

In summary, the diverse carrier systems discussed have advanced vascular stents from passive mechanical scaffolds to intelligent systems that actively modulate the vascular repair microenvironment. However, their full therapeutic potential requires precise optimization of carrier distribution, release kinetics, and local biomechanical interactions. Computational simulation technologies provide a systematic framework to address these challenges.

## 4. Computational Design of Vascular Stents

Computational simulation studies of vascular stents use computational modeling techniques to analyze stent mechanical performance, hemodynamic effects, interactions between stents and blood vessels, and therapeutic agent transport and delivery in a virtual environment, providing critical evidence for design optimization and preclinical evaluation. These computational techniques can intuitively predict the structural and mechanical responses of stents, local vascular hemodynamic changes, and targeted drug release and delivery behaviors under different stent and carrier configurations. They ultimately guide the synergistic optimization of integrated stent and carrier systems. Such computational design approaches provide a theoretical foundation for developing advanced clinical vascular stents and their personalized applications.

### 4.1. Structural Mechanics Simulation of Vascular Stents

Structural mechanics simulation employs the finite element method (FEM) to analyze the mechanical behavior of vascular stents, providing a robust theoretical foundation for stent structural optimization.

#### 4.1.1. Evaluation of Stent Mechanical Performance

Structural mechanics simulation is primarily based on finite element analysis (FEA) to quantitatively evaluate the key mechanical performance indicators of stents. The core quantifiable metrics include radial strength, elastic recoil, longitudinal foreshortening, dogboning, and bending flexibility. Radial strength, defined as the maximum external radial force a stent can withstand before collapse, is the most critical mechanical property for maintaining long-term luminal patency [24,143]. Elastic recoil and longitudinal foreshortening characterize the geometric stability of a stent after balloon deflation [143]. Excessive recoil leads to insufficient lumen gain, while significant foreshortening may result in incomplete lesion coverage or stent migration [144,145]. Dogboning refers to the non-uniform expansion phenomenon where the stent ends expand earlier and to a larger diameter than the central segment [146]. Severe dogboning can cause localized vascular wall injury [147] and insufficient stent deployment with increased residual stenosis [148]. Bending flexibility reflects the ability of a stent to navigate through tortuous blood vessels [24,149], which is essential for successful delivery and accurate deployment in complex anatomical configurations [150]. Computational simulations enable accurate prediction and systematic evaluation of these mechanical metrics prior to prototype fabrication. This predictive capability significantly accelerates the stent development process, reduces experimental costs, and provides scientific guidance for the structural optimization of vascular stents.

#### 4.1.2. Stent Structural Design and Parameter Optimization

**Figure 3:**
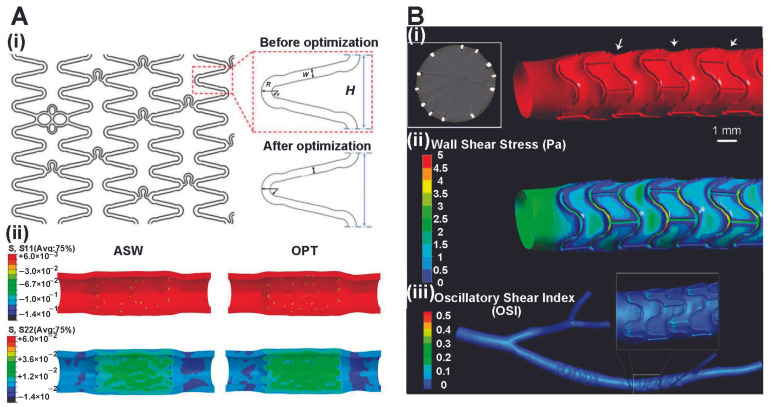
Multiphysics computational simulations for vascular stents. Multiphysics computational simulations for the rational design of vascular stents. (**A**) Geometric comparison [154]. (**i**) Geometric design parameters of the original asymmetric sinusoidal waveform and topology-optimized scaffold cells. (**ii**) Finite element analysis (FEA) contour plots demonstrating the radial and circumferential stress distributions on the vessel wall. (**B**) Hemodynamic characterization of stented coronary arteries [161]. (**i**) Micro-computed tomography imaging and 3D reconstruction of the stented arterial lumen, capturing high-fidelity stent strut geometry and identifying regions of arterial tissue prolapse (white arrows). (**ii**) Wall shear stress (WSS) distribution derived from computational fluid dynamics (CFD), highlighting low WSS regions (<0.5 Pa) in the vicinity of stent struts, which are recognized as atheroprone sites. (**iii**) Oscillatory shear index (OSI) distribution in a stented coronary bifurcation, with elevated OSI values near strut junctions indicating disturbed flow regions that correlate with atherogenesis. All images are reproduced under the Creative Commons Attribution License.

Optimizing mechanical performance through structural design is a central application of simulation-based research. Finite element analysis (FEA) allows the rapid evaluation of different design concepts without extensive physical prototyping, significantly shortening the development cycle and reducing costs [151,152]. Targeting the key mechanical metrics discussed above, systematic stent design studies have been conducted with various optimization objectives. Parametric simulation enables the systematic investigation of how core geometric parameters, including crown amplitude (A), crown radius (R), strut width (W), and strut thickness (T), influence quantifiable mechanical performance indicators, thereby establishing quantitative relationships between design variables and mechanical responses [153]. Beyond parametric tuning, topology optimization offers a more systematic approach to simultaneously reshape scaffold geometry and improve multiple mechanical properties; for example, Qiu et al. derived an optimized iron-based scaffold cell through topology optimization that achieves more uniform expansion while preserving radial strength (Figure 3A) [154]. Enhancing radial strength is one of the primary goals of structural optimization. Parametric finite element analysis can be employed to systematically examine the influence of support-ring geometry on radial strength. For example, the M-shaped bistable stent structure proposed by Xia et al., validated through simulation, overcomes the inherent limitations of conventional polymer stents, which rely on elastoplastic deformation, achieving a breakthrough improvement in radial strength [155]. The optimization of elastic recoil and longitudinal foreshortening aims to improve the geometric stability of stents after deployment. One study simulated the entire stent expansion process and found that an unequal-height support-ring design increased radial strength by more than 30% while significantly reducing elastic recoil [156]. Another study employed an optimization method integrating a Kriging surrogate model with finite element analysis, achieving a 66% reduction in recoil and a 60% reduction in foreshortening, quantitatively demonstrating improved structural deployment performance [157]. Furthermore, auxetic structures with a negative Poisson’s ratio can effectively suppress foreshortening, with an optimized stent attaining a foreshortening ratio as low as 3.27% [158]. Mitigating the dog-boning effect seeks to enhance the uniformity of stent expansion. Simulation studies have shown that dog-boning can be effectively suppressed by optimizing the end-ring geometry or connector design, thereby reducing the associated risk of vascular injury [159] as well as by adjusting the balloon length [160]. Optimizing bending flexibility requires a careful balance between radial strength and conformability. Finite element models can accurately capture the deformation response of a stent in curved vessels, guiding the rational design of connectors and the configuration of support rings. This allows for substantial improvements in bending flexibility without compromising radial strength; in one study, the bending stiffness of the optimized stent was reduced by over 30%, leading to markedly enhanced deliverability and vascular wall apposition in tortuous vasculature [159].

In summary, structural mechanics simulation has become integral to all core stages of vascular stent design. It provides a systematic reference framework for stent development, ranging from the quantitative evaluation of key mechanical performance indicators to structural parameter optimization for specific objectives. Building on the material evolution discussed in Section 2, simulation analysis can further guide the matching optimization of different materials, including cobalt-chromium alloys, magnesium alloys, and PLLA, with appropriate structural designs. For example, simulations can predict the radial support performance of high-modulus materials with thin struts, or inform the design of optimized compensatory structures for low-modulus polymers. As simulation accuracy continues to improve and multiphysics coupling methods mature, structural mechanics simulation will play an increasingly critical role in the personalized design and preclinical evaluation of vascular stents.

### 4.2. Hemodynamic Simulation of Vascular Stents

Vascular stent implantation disrupts native blood flow patterns, which is closely associated with post-procedural restenosis. Hemodynamic simulations enable quantitative assessment of these flow disturbances to guide stent design and clinical decision-making.

#### 4.2.1. Evaluation of Hemodynamics

Hemodynamic simulations primarily relies on computational fluid dynamics (CFD) methods. It quantitatively evaluates hemodynamic parameters following stent implantation [162]. Wall shear stress (WSS), oscillatory shear index (OSI), and relative residence time (RRT) serve as key hemodynamic evaluation metrics for stents.

Wall shear stress represents the hemodynamic frictional force exerted by blood flow on the vascular endothelium, serving as a critical mechanical stimulus that mediates endothelial homeostasis [163]. Studies indicate that low WSS regions are closely associated with endothelial dysfunction [163], intimal hyperplasia [164], and restenosis [165]. A value of 0.5 Pa is commonly used as the reference threshold for identifying low WSS in research [166]. Time-averaged wall shear stress (TAWSS) can be calculated by temporally averaging the instantaneous WSS over a cardiac cycle. This metric is used to evaluate the average mechanical stimulation exerted by blood flow on the vascular wall [167]. The oscillatory shear index evaluates the oscillatory characteristics of blood flow throughout the cardiac cycle. It is an important hemodynamic metric for identifying flow disturbances and predicting the risk of in-stent restenosis [168]. Studies have shown that an abnormal elevation in OSI is closely related to local blood flow disturbances following stent implantation [169]. Relative residence time integrates information from both low TAWSS and high OSI [170]. It is a critical parameter for assessing the risk of in-stent restenosis [171]. An elevated RRT value reflects an abnormal local blood flow state and correlates with near-wall flow stagnation zones [172]. This stagnant state may lead to excessive uptake of inflammatory markers by the vascular wall [173]. Furthermore, it can promote platelet aggregation and adhesion, thereby increasing the risk of thrombosis [174]. To resolve these hemodynamic metrics at the strut-level microscopic scale, compound approaches combining vascular corrosion casting, micro-computed tomography, and CFD have been developed, enabling anatomically faithful visualization of WSS and OSI distributions in stented coronary arteries (Figure 3B) [161]. Through the comprehensive analysis of these metrics, hemodynamic simulations can predict a stent’s impact on the local blood flow environment prior to implantation. This capability provides a crucial theoretical basis for stent design optimization and preclinical evaluation.

#### 4.2.2. Stent Design and Hemodynamic Optimization

The geometric design parameters of a stent, including strut thickness, cross-sectional shape, link configuration, and deployed state, significantly influence the post-implantation local hemodynamic environment [162,169]. Hemodynamic simulations enable a systematic evaluation of the impact of various design parameters on critical metrics such as wall shear stress, oscillatory shear index, and relative residence time. This evaluation provides a scientific foundation for the structural optimization of vascular stents. A teardrop-shaped streamlined cross-section can result in up to 96% of the inter-strut region maintaining a WSS above the critical threshold for in-stent restenosis. In contrast, closed-cell stents exhibit larger areas of low WSS due to a greater number of struts protruding into the lumen [175], and lower porosity can further lead to a decrease in WSS [176]. While thicker struts can reduce the expansion of low WSS regions [177], narrower strut spacing tends to increase the area of adverse low WSS [178]. Additionally, link length and its alignment angle relative to the main flow are key factors affecting WSS distribution [169]. Furthermore, factors such as malapposition, increased strut protrusion [178], and overlapping can all lead to localized hemodynamic abnormalities [171]. Regarding OSI, simulation studies indicate that strut geometry, cell configuration, porosity, link design, and the deployed state significantly impact its distribution. Stents with rounded, low-profile struts exhibit the lowest proportion of high OSI (>0.2) regions [64]. On the other hand, design features that amplify flow disturbance, such as the increased strut number in closed-cell configurations [175] and higher porosity [176], as well as connector designs with suboptimal length or alignment angle [169], all contribute to elevated OSI. Beyond the baseline design, the deployed state can further elevate local OSI in cases of malapposition or overlapping, with the adverse effect of strut size being especially amplified in overlapping configurations [171]. As a metric integrating WSS and OSI information, the distribution of RRT is notably influenced by cell configuration, strut thickness, porosity, and the deployed state. Strut thickness has been identified as the design parameter most strongly correlated with RRT [179]. The area of elevated RRT is expanded in closed-cell stents [175]. Moreover, high-porosity designs can result in elevated RRT, and overlapping regions are particularly prone to RRT elevation [180].

In conclusion, stent design parameters profoundly affect local hemodynamics in multiple dimensions. Optimizing these geometric parameters can substantially reduce adverse flow zones with low WSS, high OSI, and elevated RRT. Improving the local hemodynamic microenvironment ultimately lowers the risk of in-stent restenosis and thrombosis. Hemodynamic simulations provide vital technical support for this multi-objective optimization.

### 4.3. Drug Distribution Simulation for Vascular Stents

Vascular stents can serve as carriers for a diverse array of therapeutic agents, including small-molecule drugs, growth factors, genes, and gas molecules. By establishing mathematical models of mass transport, current simulation studies on therapeutic delivery seek to elucidate the kinetic mechanisms that govern the release of these agents from their carriers, their subsequent transport within the vascular wall, and their eventual binding to local tissues.

#### 4.3.1. Simulation of Drug Release, Transport and Target Binding

The mechanisms governing drug release from carriers are primarily categorized into three types: diffusion-controlled, degradation-controlled, and stimuli-responsive release [181]. Diffusion-controlled release is the most fundamental mode, where agent release is driven by concentration gradients in accordance with Fickian diffusion laws. Raval et al. mathematically modeled the release behavior of sirolimus from PLCL/PVP coatings, demonstrating that the process is jointly governed by surface dissolution and diffusion [182]. Zhao et al. employed a cylindrical diffusion model to quantitatively describe the everolimus release profiles from stent coatings. Their simulations revealed that a higher diffusion coefficient accelerates release, while increased coating thickness retards it. Furthermore, the in vitro diffusion coefficient was significantly higher than its in vivo counterpart, and the model-fitted thickness closely matched actual manufacturing specifications [183]. McGinty et al. investigated drug release mechanisms via analytical solutions, establishing multiple models, including pure diffusion and coupled dissolution-diffusion models. These formulations provide essential theoretical tools for estimating diffusion coefficients, dissolution rate constants, and tissue transport parameters from experimental data [184]. Degradation-controlled release involves the hydrolysis, erosion, or enzymatic degradation of the polymer matrix, with the drug release rate tightly coupled to the carrier’s degradation kinetics. Formaggia et al. pioneered a comprehensive model incorporating drug dissolution, diffusion, and surface erosion. They revealed that the relative magnitude of dissolution and diffusion rates dictates the release mode: rapid dissolution forms a moving dissolution front, whereas rapid diffusion leads to a uniform depletion of the solid phase. Additionally, surface erosion was shown to accelerate release. Based on mass conservation principles, this model also derived coupling conditions at the interface between the coating and the tissue, ensuring strict mass conservation during drug transport between the two domains [185]. Zhu and Braatz further developed a coupled mathematical model integrating PLGA coating degradation, erosion, and drug release. Their model quantified how decreasing molecular weight and increasing porosity co-regulate release behavior. Specifically, a reduction in molecular weight leads to a power-law increase in the drug diffusion coefficient within the solid polymer phase, while increased porosity creates liquid-phase diffusion pathways; together, these mechanisms synergistically modulate the release rate [186]. Additionally, Naghipoor et al. developed a non-Fickian mass transport model to systematically analyze the effects of polymer porosity and degradation rate. Their results indicated that a higher degradation rate yields a lower peak drug concentration. When time-dependent porosity was accounted for, the drug exited the stent more rapidly but exhibited a shorter residence time within the vascular wall. Conversely, a higher dissolution rate produced a higher peak concentration but a faster subsequent decay [187]. Stimuli-responsive release refers to systems where the carrier responds to physiological signals, such as pH, temperature, enzymes, or reactive oxygen species, to achieve on-demand delivery of therapeutic agents. Furthermore, utilizing finite element methods, Gagliardi analyzed the drug concentration distributions across three commercial stents, demonstrating that stent geometric design significantly influences the localized deposition of therapeutic agents within the vascular wall [188].

Binding kinetics simulations quantify the dynamic binding and dissociation processes between drugs and tissue constituents by establishing reversible reaction models. After entering the vascular wall, drugs exist partially in the free form, while the remainder binds to tissue components (e.g., receptors) to form the bound state, constituting a dynamically reversible interconversion process termed the two-phase delivery mechanism. The dynamic equilibrium between these two states jointly determines drug distribution and retention within the tissue [14]. Borghi et al. pioneered the reversible chemical reaction model to describe the interaction between hydrophilic drugs and vascular wall binding sites. This model revealed a local reservoir effect, i.e., the drug initially binds to these sites and subsequently undergoes slow dissociation, thereby prolonging its residence time within the tissue [189]. Formaggia et al. first incorporated reversible binding reactions into the arterial wall tissue module, laying the foundational framework for multi-domain coupled drug delivery models [185]. McGinty and Pontrelli further compared specific and non-specific binding models and concluded that a two-phase binding model more accurately captures the spatiotemporal distribution of drug binding to specific receptors, whereas a single-phase model is adequate for matching total tissue drug mass experiments [190]. Tzafriri et al. introduced the concept of binding potential to elucidate the threshold-dependent nature of arterial drug transport [191]. Bozsak et al. further demonstrated that sirolimus transport in the arterial wall is primarily governed by binding interactions, whereas paclitaxel transport is dominated by convective transport [86]. Saha et al. characterized the spatiotemporal dynamics of gradual binding site saturation in the vessel wall [192]. Mandal et al. identified a significant negative correlation between atherosclerotic plaque thickness and the concentration of bound drug [193]. Collectively, these binding kinetics models provide a robust theoretical foundation for understanding drug distribution and retention in arterial tissues, as well as actionable guidance for the rational design of targeted delivery strategies. Targeted delivery simulations are designed to model the active recognition process between drug carriers and specific receptors on vascular cell surfaces, enabling precise spatiotemporal drug delivery. In the field of magnetic targeting, Wang et al. developed a multi-physics coupled model to simulate the targeted delivery of drug-loaded magnetic nanoparticles to stent surfaces under an applied external magnetic field. They systematically analyzed the effects of magnetic field strength, nanoparticle size, and blood flow velocity on particle capture efficiency, providing quantitative guidance for the optimization of magnetic drug delivery systems [194]. Looking ahead, the integration of multiscale modeling approaches, from molecular recognition to macroscopic transport, holds great promise for developing comprehensive computational tools for the rational design of targeted stent coatings.

#### 4.3.2. Modulation of Drug Delivery by Carrier and Stent Design

The efficiency of stent-based drug delivery to local tissues depends not only on the physicochemical properties of the drug itself, but is also profoundly governed by the carrier structure, material characteristics, and stent geometric design. Computational simulations enable the systematic evaluation of how various design parameters influence release kinetics, tissue transport, and binding processes, thereby providing a theoretical basis for stent optimization. The choice of carrier material and its structural design largely dictate the drug release kinetics, representing a primary consideration in the design of drug delivery systems. Material type exerts a fundamental impact on release behavior. Biodegradable and non-biodegradable coatings exhibit distinctly different release profiles. By establishing simulation models, Sarifuddin et al. systematically compared the impacts of biodegradable, biostable, and polymer-free coatings on drug release, and the results showed that biodegradable coatings exhibit the most rapid drug release, whereas biostable coatings achieve a quasi-steady state (i.e., a state in which drug concentration remains relatively constant over time regardless of the degree of strut embedment), thereby demonstrating more prolonged local delivery characteristics [195]. Furthermore, the structural design of coatings offers greater degrees of freedom for modulating drug release. Sarifuddin et al. developed a computational model for bilayer coatings to elucidate the synergistic mechanism between the base and top layers, demonstrating that optimizing the kinetic parameters of both layers can significantly enhance drug delivery efficiency [196]. Porous architectures introduce an additional dimension for regulating drug release. By modeling the coating as a porous reservoir, Pontrelli et al. numerically solved the local non-equilibrium diffusion problem within porous media; their findings revealed that the delay in drug release is governed by the interplay between the physicochemical properties of the drug and the coating microstructure, thereby providing a theoretical foundation for utilizing porous carriers in drug-eluting stents [197].

Stent geometric parameters directly influence the transport pathways and spatial distribution of drugs within the tissue, representing a critical factor governing the spatial uniformity of drug delivery. This section focuses on how these geometric parameters dictate the subsequent transport behavior of therapeutic agents in the tissue. Stent geometry and connector design are primary determinants of drug release. Chen et al. found through numerical simulations that longitudinal connector designs are more effective as drug-release carriers, while excessive connector density hinders drug release [198]. Additionally, Vijayaratnam et al. utilized CFD to show that streamlined stent strut designs can simultaneously reduce coating volume and increase the coating-tissue contact area, thereby improving hemodynamics and enhancing drug uptake [199]. Inter-strut distance exerts a profound influence on the spatial distribution of drugs within the vascular wall. Mandal et al. systematically investigated this effect through numerical simulations, revealing that at small inter-strut distances, overlapping concentration profiles merge into a single broad peak. Conversely, as the distance increases, distinct concentration peaks emerge above each individual strut, highlighting inter-strut distance as a key geometric parameter for regulating spatial uniformity [200]. Furthermore, another CFD study examining curved arteries confirmed that drug deposition increases with greater inter-strut spacing [201]. The degree of compression exerted by stent struts on the vascular wall dictates the initial pathways for drug entry into the tissue. O’Connell et al. investigated idealized stent configurations and concluded that strut designs imposing less tissue compression significantly improve the spatial uniformity of drug distribution within the vascular wall [202].

While computational simulations accelerate stent optimization, their inherent limitations must be acknowledged. First, model reliability depends heavily on mesh sensitivity. Coarse meshes may underestimate mechanical strain and wall shear stress, whereas excessively refined meshes exponentially increase computational costs and preclude real-time analysis. Furthermore, models frequently rely on simplified assumptions like Newtonian blood flow. Rigorous validation against in vivo and clinical data, including 4D flow magnetic resonance imaging or intravascular ultrasound (IVUS), is therefore crucial. However, comprehensive validation is often lacking due to the scarcity of high-resolution patient data and ethical constraints. Finally, translating these simulations to routine clinical practice requires overcoming significant hurdles in data processing speed, automated image segmentation, and large-scale clinical validation. Consequently, computational findings should serve as complementary tools that guide, rather than replace, robust empirical testing.

Computational design approaches enable the intuitive and accurate prediction of structural mechanical responses, local hemodynamic states, and spatial delivery behaviors of therapeutic agents under complex parameter configurations. Through systematic evaluation of synergistic multi-factor effects and multi-objective optimization, these simulations provide a scientific basis for vascular stent design and offer valuable guidance toward personalized stent applications for individual patients in clinical practice.

## 5. Potential Clinical Applications of Vascular Stents

**Figure 4:**
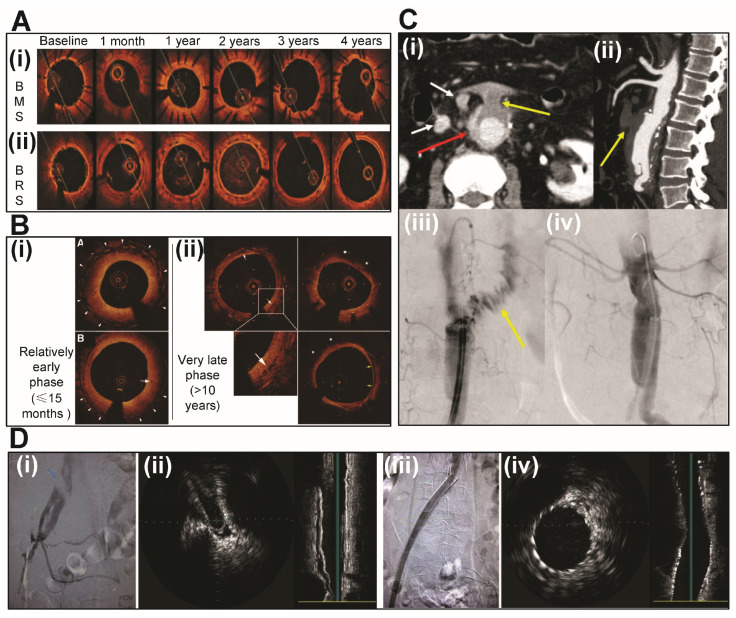
Potential clinical applications and imaging evaluation of vascular stents in different vascular beds. (**A**) Coronary artery atherosclerosis: Serial optical coherence tomography (OCT) over 4 years comparing (**i**) BMS and (**ii**) BRS. BMS maintain stable lumens; BRS enlarge (2–4 years) via remodeling and plaque regression [203]. (**B**) Peripheral artery atherosclerosis: OCT of BMS responses at (**i**) Relatively early (≤15 months) and (**ii**) very late (>10 years) phases. Relatively early phase shows peri-strut low-intensity areas (PLIA, arrowheads) and microvessels; very late phase shows neoatherosclerosis with lipid-rich intima (asterisks) and calcium (yellow arrows) [204]. (**C**) Aortic atherosclerosis: Computed tomography angiography (CTA) (**i**,**ii**) and digital subtraction angiography (DSA) (**iii**,**iv**). (**i**) Axial CTA shows infected anastomotic pseudoaneurysm (red arrow), partially thrombosed aorto-duodenal fistula (yellow arrow), and periaortic lymphadenopathy (white arrows). (**ii**) Sagittal CTA shows fistula extent (yellow arrow). (**iii**) Pre-interventional DSA confirms duodenal contrast extravasation (yellow arrow). (**iv**) Post-interventional DSA shows fistula sealed by endoprosthesis [205]. (**D**) Other vascular diseases (May-Thurner syndrome): Venography and intravascular ultrasound (IVUS). (**i**) Pre-interventional venography shows left common iliac vein compression by the right common iliac artery with collaterals. (**ii**) Pre-interventional IVUS confirms narrowed lumen. (**iii**) Post-interventional venography shows patent inferior vena cava flow. (**iv**) Post-interventional IVUS verifies optimal stent expansion, wall apposition, and venous caliber [206]. All images are reproduced under the Creative Commons Attribution License.

The clinical value of vascular stents lies in reconstructing blood flow pathways via mechanical support, thereby treating vascular stenosis, occlusion or structural abnormalities caused by diverse etiologies. Figure 4 highlights representative studies on four typical clinical applications of vascular stents [203,204,205,206]. This section provides a comprehensive review of the potential clinical applications of vascular stents. The application of vascular stents in atherosclerotic vascular disease has developed into a well-established therapeutic strategy, with its safety and efficacy validated in numerous clinical studies. With advances in materials science and interventional technology, the clinical indications of vascular stents continue to expand, providing diverse therapeutic options for the management of atherosclerotic diseases. Coronary artery atherosclerosis represents the most classic and widely utilized clinical domain for vascular stents. DES have effectively addressed the high incidence of postoperative restenosis [207], thereby significantly improving the clinical outcomes of percutaneous coronary interventions (PCI) [208] and fundamentally revolutionizing the management of coronary atherosclerosis [209]. Currently, second-generation DES with improved polymer coatings and antiproliferative agents remain the clinical standard. Meanwhile, bioresorbable scaffolds and polymer-free or fully biodegradable stent platforms are emerging as next-generation alternatives aimed at transient mechanical support and vascular restoration, but they have not yet demonstrated superior long-term outcomes compared with second-generation DES in routine clinical practice. Notably, vascular stents play a pivotal role in the treatment of both stable coronary artery disease and acute coronary syndromes. In patients with stable coronary artery disease, stent implantation is primarily performed to relieve symptoms and improve quality of life. Compared with durable polymer DES widely used in this setting, biodegradable polymer DES have shown favorable efficacy in managing small-vessel lesions [210]. Clinical research on the use of bioresorbable scaffolds in stable coronary artery disease is ongoing. Left main and multivessel disease are high-risk anatomical subsets of stable coronary artery disease [211], and studies have demonstrated that DES implantation yields sustained improvements in quality of life in patients with triple-vessel and left main disease [212]. Acute coronary syndromes (ACS) represent high-acuity clinical scenarios for stent intervention, comprising ST-segment elevation myocardial infarction (STEMI), non-ST-segment elevation myocardial infarction (NSTEMI), and unstable angina (UA) [213]. In patients with STEMI, primary PCI serves as the cornerstone reperfusion strategy. Clinical studies have shown that BRS technology, through optimized design combined with intravascular imaging guidance, can achieve complete resorption without permanent metallic implants, effectively mitigating thrombotic risk while preserving physiological vasomotor function [203]. For patients with STEMI, implantation of new-generation DES significantly improves long-term prognosis [214]. Similarly, stent implantation constitutes a cornerstone therapy for patients with UA. Peripheral artery atherosclerosis represents a major application domain for vascular stents, with femoropopliteal, infrapopliteal, and iliac artery disease constituting typical anatomical subsets [215]. Critical limb ischemia represents a leading cause of amputation, wherein stent implantation is indicated to achieve limb salvage and optimize patient prognosis [216]. Infrapopliteal artery atherosclerosis remains a significant therapeutic challenge, which is particularly prevalent in patients with diabetes mellitus [217]. A meta-analysis demonstrated that bioresorbable scaffolds implanted in infrapopliteal arteries achieved a 1-year primary patency rate of approximately 90% and a freedom from target lesion revascularization (TLR) of 96%, indicating favorable short-term clinical outcomes [218]. Concurrently, femoropopliteal lesions are exposed to complex dynamic mechanical stresses, including bending, compression, and torsion, which contribute to a relatively high rate of stent fracture [219]. DES technology has significantly enhanced therapeutic efficacy for femoropopliteal disease, with data showing a 1-year clinical patency rate of 84% and a freedom from TLR of 89%, thereby confirming satisfactory postprocedural recovery [220]. For iliac artery atherosclerosis, endovascular stenting yields excellent outcomes and represents a highly mature clinical domain. The SENS-ILIAC randomized controlled trial demonstrated a 1-year clinical patency rate of up to 99% for iliac artery stenting, confirming its role as a highly effective intervention for atherosclerotic occlusive disease [221].

Covered stent grafts represent a critical therapeutic modality for conditions associated with aortic atherosclerosis. Numerous studies have confirmed favorable clinical outcomes after stent deployment in patients treated with these devices. The pathogenesis of abdominal aortic aneurysms and certain types of aortic dissection is closely associated with aortic atherosclerosis [222,223]. Studies have shown that patients with ruptured abdominal aortic aneurysms treated with covered stent grafts exhibit significantly lower 30-day mortality compared with those undergoing open surgery [224]. For complex dissections involving the aortic arch and descending thoracic aorta, the frozen elephant trunk technique combined with stent implantation offers a novel surgical alternative [225].

In summary, managing atherosclerotic diseases across these distinct anatomical domains necessitates integrating computational design with targeted drug delivery. Coronary interventions rely on optimized hemodynamics and precise drug release kinetics to prevent thrombosis and restenosis. Conversely, the dynamic biomechanics of peripheral arteries require finite element analysis to mitigate stent fracture, while complex aortic pathologies depend on patient-specific modeling to optimize stent-graft deployment.

Beyond atherosclerotic vascular disease, vascular stents have also demonstrated favorable therapeutic efficacy across a diverse spectrum of other disorders, including inflammatory vascular diseases and vascular compression syndromes. This section provides a comprehensive review of these clinical applications. Inflammatory vascular diseases, such as Takayasu arteritis [226] and cerebral vasculitis [227], can lead to vascular stenosis and occlusion. When medical therapy is ineffective, stent implantation can serve as a salvage treatment. A published case report has demonstrated that a patient with Takayasu arteritis remained free of restenosis or reocclusion at 2-year follow-up after stent implantation, indicating that vascular stent therapy can yield a favorable prognosis during the stable phase of inflammation [228]. For cerebral vasculitis refractory to medical treatment, stent implantation provides a salvage treatment option, and its combination with intensive medical therapy has demonstrated favorable clinical outcomes [229]. Vascular compression syndromes represent a group of disorders caused by the extrinsic compression of blood vessels by surrounding anatomical structures, including May-Thurner syndrome and nutcracker syndrome [230]. May-Thurner syndrome is effectively managed with iliac vein stent implantation. Compared with balloon angioplasty alone, stent placement effectively resists the persistent mechanical compression from the overlying right common iliac artery. Furthermore, intraoperative guidance using intravascular ultrasound (IVUS) allows precise assessment of the degree of compression and optimizes stent sizing and positioning, thereby improving long-term primary patency rates [206]. Similarly, nutcracker syndrome is also amenable to endovascular stent placement. In a recent series of patients treated with either surgical transposition or stenting, the overall 1-year primary patency rate was 87%, with symptom relief rates exceeding 80% for both flank pain and hematuria [231]. Furthermore, vascular stents play a critical therapeutic role in the management of vascular injuries, congenital vascular malformations, and post-transplantation vascular complications. For vascular injuries, stents are particularly useful for managing iatrogenic injuries, including iliac artery occlusion after laparoscopy [232], arteriovenous fistulas following lumbar surgery [233], and high-output heart failure caused by arteriovenous fistulas after lumbar disc surgery [234]. For congenital vascular malformations, stenting is a key interventional modality, especially in pediatric patients, as it can delay or obviate the need for complex open surgery. Successful stent implantation has been reported in multiple conditions, including coarctation of the aorta [235], branch pulmonary artery stenosis after complex congenital heart disease repair [236]. For post-transplantation vascular complications, stents offer a minimally invasive salvage therapy. Complications including transplant renal artery stenosis [237], and hepatic artery stenosis after liver transplantation [238].

Although these non-atherosclerotic conditions present diverse etiologies, they increasingly demand tailored computational and pharmacological innovations. Specifically, the persistent extrinsic mechanical stresses in compression syndromes necessitate finite element analysis to optimize stent radial strength. Meanwhile, complex congenital or post-transplantation anatomies rely on patient-specific computational modeling, and refractory inflammatory vasculitis highlights the potential for stents delivering localized immunosuppressive therapies.

The clinical applications reviewed span a broad spectrum of vascular diseases. Across all these clinical scenarios, vascular stents have consistently demonstrated therapeutic value beyond mechanical support, encompassing drug delivery, local pathology management, and minimally invasive vascular reconstruction. Nevertheless, challenges remain in achieving personalized design, reliable long-term outcome prediction, and precise spatiotemporal control over drug delivery. The following section explores how artificial intelligence can address these challenges and usher in a new era of intelligent, personalized vascular interventions.

## 6. Future Perspectives in Vascular Stent Development

### 6.1. Artificial Intelligence and Vascular Stent

The core missions of vascular stents encompass restoring vascular integrity, optimizing hemodynamics, and providing therapeutic functionality through controlled drug delivery. Together, these functions determine their clinical efficacy of stent-based interventions. With the rapid advancement of artificial intelligence, its profound integration across materials development, carrier design, and optimization via simulation is paving new pathways toward the ultimate realization of precision clinical decisions.

Serving as a comprehensive theme, artificial intelligence is systematically reshaping the entire lifecycle of vascular stents, fundamentally transitioning the field from traditional experimental approaches to methodologies guided by data.

### 6.2. Intelligent Design for Vascular Stent

Driven by AI, the intelligent design of vascular stents is currently manifesting across multiple dimensions.

In the realm of stent materials development, as discussed in preceding sections, biodegradable metallic stents face the challenge of balancing degradation kinetics, while polymeric stents are constrained by inherent mechanical limitations. Data-driven strategies are therefore emerging as powerful tools to address these long-standing challenges. By constructing quantitative relationships among material composition, processing parameters, and performance, machine learning enables rapid screening of optimal formulations from large compositional spaces thereby shifting the paradigm of alloy stent design from empirical exploration to predictive precision [239,240]. This data-driven methodology has been successfully extended to titanium alloys and other metallic material systems, significantly expanding the design space for intelligent vascular stent materials [241,242]. Recently, the integration of deep learning with high-resolution imaging enables quantitative characterization of the in vivo stent degradation behavior, providing a novel evaluative tool for the precise modulation of degradation [243]. Lattice superstructures, a novel architectural configuration for metallic stents, have been optimized through machine learning-assisted design to achieve synergistic regulation of mechanical performance and superelasticity, thereby introducing a new paradigm of integrated structural-functional design for next-generation high-performance vascular stents [244]. In the polymeric domain, the application of machine learning to the optimization of polylactic acid properties has attracted increasing attention; by enhancing mechanical performance and refining processing parameters, novel strategies for the design of bioresorbable polymeric scaffolds are emerging [245]. Furthermore, the convergence of artificial intelligence with additive manufacturing is empowering the entire chain of stent fabrication, spanning compositional design, structural optimization, and process control [246]. It can be anticipated that, as data-driven methodologies become deeply integrated with materials science, artificial intelligence will accelerate the resolution of critical bottlenecks, including insufficient control over alloy stent degradation kinetics and inadequate mechanical support of polymeric stents, ultimately transforming on-demand design into a tangible reality. Achieving this goal, however, will depend not only on the powerful predictive capacity of machine learning but also on the development of interpretable models capable of elucidating the intrinsic relationships between material parameters and performance outcomes [245]. Such interpretability will facilitate the inverse design of optimal material formulations and architectures tailored to specific clinical requirements, including predetermined support duration, degradation profiles, and mechanical demands, thereby providing an essential reference framework for the development of next-generation biodegradable, high-performance vascular stents.

In the realm of carrier functional design, hydrogels, fibers, nanoparticles, microspheres, and other carriers endow vascular stents with multiple biological functions, including anti-proliferative, anticoagulant, and pro-endothelialization activities. Recent research has demonstrated a distinct trend from single-agent release toward multi-drug synergistic delivery, and from passive release toward targeted delivery, while temporally regulated strategies are being actively explored in cutting-edge domains such as gas-based therapeutics. The integration of artificial intelligence is accelerating this paradigm shift. Specifically, regarding intelligent carrier design and screening, machine learning can assist in designing stimuli-responsive materials and optimizing the mechanical properties, degradation kinetics, and release profiles of hydrogels, thereby opening new avenues for personalized, on-demand release strategies in the design of stent coating carriers [87].

In the realm of biomimetic carriers and smart structures, the convergence of AI with nanoengineering, 4D printing, and other advanced technologies is creating carrier systems with dynamic and stimuli-responsive functionalities. Biomimetic carriers, exemplified by exosome-mimetic nanovesicles, can achieve more precise targeted delivery and immunomodulation, while the integration of artificial intelligence-assisted design offers new opportunities for their personalized application and optimization [247]. In terms of carrier performance evaluation, machine learning is substantially enhancing computational efficiency. Artificial intelligence technologies can be deployed across the entire lifecycle of vascular stent carrier, thereby demonstrating the potential for end-to-end empowerment from carrier design to clinical application [248]. As artificial intelligence becomes deeply integrated with carrier engineering, vascular stent carriers are rapidly evolving from passive release platforms toward intelligent responsive systems. This transformation relies not only on the precise prediction of carrier material properties through machine learning but, more critically, on its capacity to uncover the complex relationships among carrier architecture, release behavior, and biological responses. It is anticipated that in the near future, intelligent carrier systems with programmed release kinetics can be inversely designed according to the temporal demands of vascular injury repair, thereby providing pivotal support for the development of next-generation personalized, temporally sequenced vascular therapeutics.

In the realm of personalized stent design, the convergence of artificial intelligence with patient-specific data is opening new frontiers. Deep learning models trained on medical imaging data can rapidly reconstruct patient-specific vascular anatomies and automatically identify lesion characteristics, such as the degree of calcification and plaque composition, thereby providing a precise basis for stent selection and implantation strategies [249]. Furthermore, frameworks integrating finite element simulation with machine learning can predict the deployment configuration and mechanical compatibility of various stent designs in patient-specific vascular anatomies [250], enabling interventionalists to preoperatively simulate procedural outcomes and select optimal stent configurations through virtual surgical planning platforms tailored to patient-specific anatomy. Moreover, the fusion of digital twin technology with artificial intelligence holds promise for enabling real-time intraoperative feedback. For the interventional treatment of coronary bifurcation lesions, a framework combining hemodynamic simulation with machine learning can provide real-time assessment of functional flow indices of the side branch following stent implantation, thereby reducing periprocedural uncertainty for each patient [251]. Underpinning these specific technologies is the integrated application of surrogate models, shape optimization, and patient-specific design methodologies, which together constitute a methodological framework for the rapid development of next-generation personalized stents [252]. Through the deep mining of patient-specific data and real-time functional assessment, artificial intelligence is propelling vascular stent interventions from population-based protocols toward individualized decision-making, better addressing the unique clinical needs of each patient and providing essential references for the personalized design of vascular stents and intraoperative clinical decision-making.

In the realm of prognostic prediction and risk stratification, artificial intelligence is transforming clinical assessment from post hoc evaluation to proactive early warning. Machine learning models based on multimodal data, including medical imaging, biochemical markers, and clinical characteristics, can predict the risk of post-stenting complications such as restenosis and thrombosis, providing decision support for personalized anticoagulation and follow-up regimens [253,254]. Furthermore, previous studies have demonstrated that machine learning models based on imaging-derived digital twins can effectively predict the risk of endoleaks after vascular stent implantation [255]. At present, the integrated application of AI across key stages including design optimization of drug-coated cardiovascular devices, real-time imaging interpretation guidance, and postoperative complication prediction is demonstrating significant potential for end-to-end empowerment from bench to bedside [248]. Collectively, these technologies are driving the integration of risk warning throughout the entire patient treatment cycle, spanning preoperative planning to postoperative follow-up, thereby delivering comprehensive clinical care support.

### 6.3. Conclusion for Future Perspectives in Vascular Stent

In conclusion, artificial intelligence is comprehensively reshaping the research, development, and application paradigms of vascular stents across materials science, carrier design, simulation optimization, clinical decision-making, and prognostic management. The on-demand design of materials, intelligent responsiveness of carriers, intelligent prediction of simulations, individualized decision-making in clinical practice, and comprehensive early warning in prognosis are interwoven and evolving synergistically, collectively propelling vascular stents into a new era of intelligence and precision. With the deep integration of artificial intelligence with materials science, carrier technology, biomechanics, and clinical medicine, vascular stents will gradually achieve integrated intelligent design that unifies structure, function, and therapeutic efficacy. In the future, research efforts are expected to rapidly iterate optimal vascular stent design solutions in virtual environments based on individual patient characteristics, and further advance the realization of real-time intraoperative adjustment of interventional strategies, thereby bringing safer, more precise, and more personalized therapeutic prospects to patients with cardiovascular diseases.

## Figures and Tables

**Table 1:** Classification, core characteristics and main limitations of vascular stent materials.

Material Classification	Representative Materials	Core Characteristics	Main Limitations
Non-degradable Metals	Stainless Steel	First stent material, excellent mechanical properties, low cost	High ISR rate, permanent retention
	Cobalt-chromium Alloy	High strength and radiopacity, allows for thin-strut stents ~300–600 MPa	Risk of nickel ion allergy, permanent retention
	Platinum-chromium Alloy	Combines high strength and good radiopacity	Permanent retention
	Nickel-titanium Alloy (Nitinol)	Superelasticity, shape memory, self-expanding	Potential toxicity of nickel ions, permanent retention
Biodegradable Metals	Magnesium Alloy	First commercial biodegradable metal stent; elastic modulus ~40–45 GPa; mechanical properties superior to polymers	Degrades too fast, hydrogen gas production
	Iron Alloy	Excellent mechanical properties (close to stainless steel)	Degrades too slowly
	Zinc Alloy	Moderate degradation rate; degradation time ~1–2 years	Mechanical properties need improvement
Biodegradable Polymers	Polyesters	Fully degradable, tunable degradation rate (6 months to >2 years)	Insufficient radial support, lower mechanical properties than metals
	Polyurethanes	Smart responsiveness, excellent biocompatibility	Long-term stability remains to be verified
	Polycarbonates	Inherent X-ray opacity, radial strength ~20–40% higher than PLLA systems	Long degradation period (36–48 months)

**Table 3:** Comparison of antiproliferative agents in stent-based drug delivery.

Feature	Sirolimus (Limus Family)	Paclitaxel
Mechanism of action	Inhibits mTOR, G1 → S phase arrest	Stabilizes microtubules, M phase arrest
Effect type	Cytostatic (anti-proliferative, non-apoptotic)	Cytotoxic (apoptosis-inducing)
Target protein	FKBP12	Microtubules
Transport dominance	Binding-dominated	Convection-dominated
Lipophilicity	High	Very high
Anti-inflammatory effect	Yes	No
Endothelial healing	Faster	Slower

## Data Availability

No new data were created or analyzed in this study.

## Abbreviations

Acute Coronary Syndromes	ACS
Artificial Intelligence	AI
Activated Partial Thromboplastin Time	APTT
Basic fibroblast growth factor	bFGF
Bare-Metal Stent	BMS
Bioresorbable Scaffold	BRS
Computational Fluid Dynamics	CFD
Cobalt-Chromium	Co-Cr
Computed Tomography Angiography	CTA
Dual Antiplatelet Therapy	DAPT
Drug-Eluting Stent	DES
Digital Subtraction Angiography	DSA
Drug Docetaxel	DTX
Endothelial Nitric Oxide Synthase	eNOS
Finite Element Analysis	FEA
Finite Element Method	FEM
Fibroblast Growth Factor	FGF
In-Stent Restenosis	ISR
Intravascular Ultrasound	IVUS
Late Stent Thrombosis	LST
Nitric Oxide	NO
Non-ST-Segment Elevation Myocardial Infarction	NSTEMI
Optical Coherence Tomography	OCT
Oscillatory Shear Index	OSI
Percutaneous Coronary Intervention	PCI
Polycaprolactone	PCL
Poly(Lactic-co-Glycolic Acid)	PLGA
Poly(L-Lactic Acid)	PLLA
Platinum-Chromium	Pt-Cr
Polyvinyl Alcohol	PVA
Relative Residence Time	RRT
Reactive Oxygen Species	ROS
Scanning Electron Microscopy	SEM
Smooth Muscle Cell	SMC
Stainless Steel	SS
Stent Thrombosis	ST
ST-Segment Elevation Myocardial Infarction	STEMI
Time-Averaged Wall Shear Stress	TAWSS
Target Lesion Revascularization	TLR
Thrombin Time	TT
Unstable Angina	UA
Vascular Endothelial Growth Factor	VEGF
Wall Shear Stress	WSS
